# Supplementary material for: FERN – a Java framework for stochastic simulation and evaluation of reaction networks
Source: BMC Bioinformatics. 2008 Aug 29;9:356. doi: 10.1186/1471-2105-9-356 (PMC2553347; doi:10.1186/1471-2105-9-356)
Supplement: Additional file 1 — FERN distribution, Version 1.3. This archive contains the FERN source code and binaries as well as documentation and example models in FernML and SBML. [file 1471-2105-9-356-S1.zip › fern/doc/javadoc/fern/tools/gnuplot/class-use/ArrayMatrixAxes.html]

Uses of Class fern.tools.gnuplot.ArrayMatrixAxes


---


|  |  |  |
| --- | --- | --- |
| **Overview** \| **Package** \| **Class** \| **Use** \| **Tree** \| **Deprecated** \| **Index** \| **Help** | |  |
| PREV   NEXT | **FRAMES**    **NO FRAMES**     **All Classes** |  |


---


## **Uses of Class fern.tools.gnuplot.ArrayMatrixAxes**

| Packages that use ArrayMatrixAxes | |
| --- | --- |
| **fern.tools.gnuplot** | Provides classes to use gnuplot from java. |

Uses of ArrayMatrixAxes in fern.tools.gnuplot

| Subclasses of ArrayMatrixAxes in fern.tools.gnuplot | |
| --- | --- |
| `class` | `TransposedArrayMatrixAxes`             Implementation of `Axes` to use with transposed array matrixes of any number type (the type of the arrays is inferred by using the reflection api). |

---


|  |  |  |
| --- | --- | --- |
| **Overview** \| **Package** \| **Class** \| **Use** \| **Tree** \| **Deprecated** \| **Index** \| **Help** | |  |
| PREV   NEXT | **FRAMES**    **NO FRAMES**     **All Classes** |  |


---
